# An examination of the effects of family, school, and community resilience on high school students’ resilience in China

**DOI:** 10.3389/fpsyg.2023.1279577

**Published:** 2024-01-11

**Authors:** Chunlin Qi, Nanchang Yang

**Affiliations:** College of Education, Jiangxi Normal University, Nanchang, China

**Keywords:** family resilience, school resilience, community resilience, individual resilience, resilience

## Abstract

**Introduction:**

Resilience plays a pivotal role in shaping the academic accomplishments, psychological well-being, and future prospects of high school students. Despite its significance, there is a notable dearth of studies examining the current state and determinants of resilience among high school students in China.

**Methods:**

This research addresses this gap by assessing and exploring levels of resilience and their interconnections across four key domains—individual, family, school, and community—among Chinese high school students. The study also investigates variations in resilience based on factors such as gender, geographical location, and grade levels. A total of 667 high school students participated in the study, responding to four resilience scales.

**Results:**

Chinese high school students exhibited generally low levels of resilience, with family resilience ranking the highest and community resilience the lowest. The study revealed that resilience is positively influenced by resilience levels in school, family, and community settings. Consequently, strategies aimed at fortifying resilience should prioritize interventions in familial, educational, and communal environments.

**Discussion:**

Moreover, the research findings indicate noteworthy disparities in resilience among high school students based on gender, urban-rural divide, and grade levels. Female, urban, and lower-grade students displayed higher resilience compared to their male, rural, and higher-grade counterparts. This highlights the importance of focusing on resilience-building measures tailored to male, rural, and higher-grade high school students, given their increased likelihood of facing significant challenges and stressors in both academic and personal spheres.

**Conclusion:**

This study contributes to the broader understanding of resilience by investigating the correlations between individual, family, school, and community resilience among Chinese high school students. The findings underscore the need for targeted interventions to enhance resilience, particularly in specific demographic groups, thereby advancing the efficacy of resilience-building techniques in high school settings.

## 1 Introduction

During high school, adolescents undergo a critical phase in their lives. A multitude of stresses and challenges characterize this time. Along with physical and emotional changes, students encounter more challenging academic work. They struggle with the contradictory feelings that arise while shifting from childhood to adulthood. They establish new relationships and experience the enchantment of first love. Adding to their stress is the tremendous pressure of taking college admission examinations. In addition, substantial cognitive and emotional development occurs during high school, which may result in psychological disagreements and challenges adjusting to novel situations. The challenges that stem from these concerns encompass heightened susceptibility to stress, heightened vulnerability to setbacks, a tendency toward submissiveness in the face of adversity, and a proclivity to withdraw from challenging situations. These issues can significantly affect their mental health and potentially contribute to the emergence of psychiatric disorders (Lin and Yusoff, 2013). Multiple publications have shown instances of high school students experiencing boredom with school, encountering parental conflicts, running away from home, and even engaging in self-harm or suicide as a result of the enormous pressure and psychological difficulties they confront (Kuftyak, 2015; Moore and Woodcock, 2017; Zhang et al., 2020; Anderson et al., 2022). Ultimately, all of these problems can be attributed to their inherent psychological vulnerability and absence of cognitive resilience. Societal emphasis is growing on the significance of children and adolescents cultivating psychological resilience, resistance, and the capacity to surmount adversity. Character education and the resilience of high school students have emerged as important areas of research in core literacy (White, 2010; Hinduja and Patchin, 2017). Therefore, examining and taking action on individual resilience among high school students is imperative. This can contribute significantly to their strength and nurture the comprehensive advancement of their psychological health.

Resilience, in the realm of psychology, is commonly regarded as an individual attribute associated with qualities such as fortitude, flexibility, problem-solving acumen, intellect, a capacity for humor, and social aptitude (Iarocci et al., 2009). Resilience is influenced by individual characteristics and broader aspects at the systemic, social, and community levels (Kirmayer et al., 2009). Adaptability, reactivity, and tenacity among the ties that comprise a family, school, community, or more extensive social network constitute systemic resilience. This is because individual resilience cannot exist alone. Everyone is affected by their immediate social circle, upbringing, and surroundings. Individuals’ cultural values and views are shaped by their socialization. Hence, interventions that prioritize family, school, and social resilience are essential for cultivating individual resilience. The role of social support, family, and schools as stress buffers has received much attention (Ringdal et al., 2020), but the interplay between these networks and the development of resilience as an individual has received less consideration.

Although there is enough understanding of the significance of family members as a means of social support and safeguarding, there needs to be more investigation into the contribution of schools and community environments in assisting adolescents (Dryfoos et al., 2005). According to Dolan (2011), there is contention among specialists that children and adolescents can enhance their psychological health and resilience by receiving assistance from several channels, including their household, educational institution, and local community. An individual, family, community, and society can become more resilient through psychological and social adjustments and adaptations (Kirmayer et al., 2011).

The study on the resilience of high school students has several limitations. There needs to be more comprehension of the impact of individual resilience on high school students in relation to family, school, and community resilience. The reason behind this is that high school students’ resilience can be shaped by a multitude of elements, both internal and external, including their family, school, and community. Consequently, it becomes arduous to comprehensively analyze all these factors to investigate high school students’ fortitude. Furthermore, the existing literature on high school children’s resilience needs to be revised. In recent years, there has been a surge in research focusing on high school students’ psychological and academic resilience. This research has investigated various vital considerations, including academic self-efficacy (Mao et al., 2023), subjective wellbeing (Meng et al., 2023), academic stress (Choi et al., 2023), and academic anxiety (Fiorilli et al., 2020).

The primary objective of our study is to assess the present level of resilience among Chinese high school students, with a specific emphasis on individual, family, school, and community resilience. In addition, our objective is to examine the role of family, school, and community resilience in fostering individual resilience in Chinese culture. In addition, our research endeavors to contribute to an extensive database and offer theoretical direction to enhance resilience among high school students. Moreover, our objective is to fill the existing research void on the impact of family, school, and community resilience on individual resilience, thus broadening the domain of resilience research. In order to achieve these goals, our research aims to address the following inquiries:

(1) What is Chinese high school students’ individual, family, school, and community resilience?

(2) What is the correlation between the resilience of Chinese high school students at the individual, family, school, and community levels?

## 2 Literature review

### 2.1 Individual resilience

Individual resilience combines personal qualities, activities, and mindsets that contribute to one’s overall physical, emotional, and social wellbeing (Bohman et al., 2017). The capacity to avert exhaustion and adeptly manage challenging circumstances or intense pressure is encompassed within it (Mackay, 2003). Individual resilience is a complex and multifaceted idea that differs based on geographical location, context, and specific adversity types. Research undertaken during the COVID-19 pandemic in Israel, the Philippines, and Brazil discovered that variations in individual resilience between nations can be primarily attributed to psychological and ecological variables (Ballada et al., 2022). Furthermore, a study conducted to determine crucial factors contributing to an individual’s ability to cope with the challenges posed by the COVID-19 pandemic revealed a substantial positive correlation between age, education level, and individual resilience (Ferreira et al., 2020). Conversano et al. (2020) presented a paper that discussed the factors contributing to psychological distress among healthcare professionals during the COVID-19 pandemic. The study identified adequate social support, self-efficacy, internal locus of control, and coherence as individual resilience factors. In education, individual resilience is characterized as a quality that enables children and adolescents to achieve academic success despite encountering obstacles, adversities, and other hardships in life (such as mental health issues, academic stress, financial difficulties, or other unfavorable circumstances). Mental health and wellbeing are promoted by individual resilience, especially in children and adolescents (Basu et al., 2020).

Individual resilience exhibits the following traits: It encompasses multiple dimensions, including mental, emotional, behavioral, physiological, and social components, each of which might have unique influence and manifestation (Masten et al., 1990). Individual resilience is not a fixed trait but an evolving process affected by the dynamic interplay between individuals and their surroundings (Henshall et al., 2020). Resilience is not innate but rather a skill that people can develop through experience and instruction (Laird et al., 2019). Individual resilience is not a one-size-fits-all trait but rather one that is situationally dependent; hence, one’s resilience tactics may need to change depending on the circumstances (Ungar, 2011). Various goals may have various resilience criteria; hence, resilience is not an end but a purpose associated with adaptation and development (Masten, 2007). Furthermore, individual resilience is shaped by many personal and contextual elements, including personality traits, beliefs, attitudes, emotions, skills, resources, support systems, and opportunities. Various elements, including the family, school, and community, can foster individual resilience. Family resilience is crucial to improving individual resilience in low- and middle-income countries with limited resources (Basu et al., 2020). Furthermore, research has demonstrated that school-based treatments, namely life skills education, enhance individual resilience (Basu et al., 2020; Nilsson et al., 2023). A qualitative study demonstrated that several personal and environmental elements, such as neighborhood, family, school, and personal and peer experiences, substantially influenced children’s resilience (Abbott, 2014).

### 2.2 Family resilience

The notion of family resilience, derived from examining individual resilience, is increasingly acknowledged. Family resilience pertains to the capacity of a family to adeptly manage and surmount difficulties and pressures constructively. The primary objective is to guarantee the seamless integration of children, adolescents, and young individuals into society. The adaptive capacity of a family system is its capability to endure or recuperate from any challenge that threatens to disrupt its progress or stability. Walsh (2015) frequently refers to this concept as “family shock absorbers.” Families do not exist exclusively in a condition of either being resilient or not being resilient; instead, they demonstrate different degrees of resilience depending on the particular stressors they encounter. Prior studies have examined the positive aspects of families and their ability to cope with stress. However, it was not until the 1990s that researchers in the field of family science began using the phrase “family resilience.” Herdiana et al. (2018) found that family resilience significantly impacts individual resilience, highlighting the vital role of the family in fostering resilience among high school students. From a family systems viewpoint, it is recognized that families operate as autonomous systems that engage with other systems, such as educational institutions, communities, and social or ecological systems. To ensure equilibrium and coherence, families must possess the ability to adapt their roles, objectives, principles, regulations, and priorities through external modifications (Patterson, 2002). Resilience plays a crucial role in forming families, enabling them to effectively navigate challenges and discover strategies to manage, accommodate, and even flourish (Roncaglia, 2019). Conversely, families who lack resilience are more prone to surrendering or being overwhelmed. Family resilience fosters a secure, adaptable, and nurturing atmosphere that encourages solid familial bonds and enables favorable growth and maturation in adolescents.

The concept of family resilience extends beyond perceiving individual family members as potential providers of individual resilience. The primary focus is examining the potential hazards and the ability to recover the family as a cohesive entity. The fundamental principle of this system perspective is that significant crises and ongoing adversity affect the entire family unit. Critical family processes play a role in either facilitating or hindering the adaptability of all family members and the family. The family’s reaction is crucial. Significant stressors can disturb the operation of the family system, causing consequences that extend to all members and their interpersonal connections. The main mechanisms of resilience empower family structures to reorganize during periods of crisis, alleviate stress, decrease the likelihood of dysfunction, and facilitate optimal adjustment (Walsh, 2011). Contrary to the emphasis on individual resilience, family resilience examines the collective ability of a family to adjust and react to stress. Consequently, the collective effect of the family might impact an individual’s capacity to handle difficulties. Family resilience is developed by accumulating protective factors within the family and responding positively to challenging circumstances (Simon et al., 2005). Family protective factors encompass various elements that contribute to the wellbeing and resilience of a family unit. These factors include engaging in collective problem-solving, maintaining optimistic mindsets, demonstrating flexibility and adaptability, fostering effective communication, practicing honesty and pragmatism, promoting equality, prioritizing physical and mental health, and seeking social support (Bethell et al., 2016). Each of these characteristics has a role in cultivating individual resilience within the family unit.

### 2.3 School resilience

School resilience has a significant impact on the resilience of both teachers and students. It aids individuals in surmounting challenges and adjusting to adverse circumstances during catastrophic events. Sociologically speaking, school resilience refers to the capacity of school members to effectively adapt and recover in the wake of a crisis. Schools play a crucial role in lessening the impact of catastrophes due to their ability to address and resolve problems during and after a disaster effectively. In addition, it is the social obligation of educational institutions to foster a culture of catastrophe awareness and enhance the resilience of their members (Ungar et al., 2014). School resilience can be examined from various perspectives, including the individual and broader school system levels (Mackay, 2003). The primary concern in this scenario is the overall resilience of high school students. In this sense, school resilience refers to the ongoing process through which high school students effectively adapt to the difficulties they encounter in the educational environment. These concerns have consequences not only for students’ academic performance but also for their psychological and physical wellbeing. Fortunately, schools possess the capacity to establish a supportive atmosphere for high school adolescents, which is essential for cultivating resilience. Through education on resilience, schools can enhance the mental wellbeing of youngsters, cultivate good emotions, and enable them to live more satisfying lives (Becvar, 2013). Multiple research studies have demonstrated that schools can safeguard children and adolescents, aiding them in managing the diverse difficulties they face in a demanding world (Bryan and Henry, 2008).

### 2.4 Community resilience

Community resilience refers to a community’s capacity to bounce back from significant changes, sustain its ability to adjust, integrate knowledge gained from crises, and facilitate future development. It encompasses the elements of social capital, physical infrastructure, and deeply rooted patterns of interdependence. Community resilience can be conceptualized in two primary dimensions: Firstly, it examines how individuals utilize social networks and cultural resources to cope with stress and challenges effectively. Secondly, it encompasses the collective responses of communities to stress and challenges aimed at restoring their functioning and showcasing their resilience (Grazia and Molinari, 2021). Promoting health and wellbeing relies on community resilience, and interventions can enhance the quality of families and communities and individual resilience (Basurto-Cedeño and Pennington-Gray, 2018).

Examining the fundamental factors contributing to individual resilience might provide insight into how community resilience fosters and amplifies individual resilience. An analysis can be conducted on the multiple aspects that contribute to an individual’s resilience and how they are connected to the structures and processes within the community. This relationship can enhance, bolster, and reinforce individual-level characteristics. The composition of age, gender, and race in social networks within a community might impact the characteristics and benefits of those networks (Harvey, 2011). The community’s resilience is contingent upon society’s response to problems that have the potential to disrupt or endanger the community. This response pertains to the process of individuals, groups, and organizations modifying and accommodating themselves to the community and the community’s engagement with its environment, encompassing other social, economic, and political entities. Moreover, the existence of a community and other contextual factors can significantly impact an individual’s physical and mental health and behaviors. For example, the widespread presence of fast food and processed food choices in the neighborhood and the limited access to fresh produce impact an individual’s dietary preferences, nutritional intake, and overall wellbeing. The communal environment in which individuals reside undeniably influences their employment, education, and overall lives, exerting positive and negative effects.

Research on community resilience has revealed a high correlation between the achievement of individuals and the prosperity of their entire community. A community’s prosperity is contingent upon its resources, encompassing both informal social support systems for individuals and formal social service systems such as child welfare, education, corrections, and healthcare (Ungar, 2011). This concept of community resilience highlights a change in viewpoint, shifting the focus from a community’s vulnerabilities to recognizing its capacity to utilize its existing resources efficiently. Individual resilience is intricately connected to the resilience of others in the community within multi-faceted community resilience.

Based on prior research examining individual resilience as well as resilience within families, schools, and communities, this study aims to suggest the following hypotheses:

H1: Resilience in families, schools, and communities is positively associated with resilience in high school students.

## 3 Theoretical background

Merely examining resilience as an individual trait is inadequate; it is more advantageous to examine resilience within the individual as a subsystem within larger systems, encompassing the individual’s resilience in the family, school, community, and even society. This aligns with Bronfenbrenner’s ecosystem hypothesis (Mwangi et al., 2017) and the multi-system resilience theory (Ungar et al., 2021). Bronfenbrenner’s model is a conceptual framework that provides insight into human development (Frankel, 1992). In Bronfenbrenner’s ecosystem theory, resilience arises from the dynamic character of interactions within a specific ecological setting. Ecological models define resilience as a process at various levels: micro-system, meso-system, exo-system, and macro-system. The study uses Bronfenbrenner’s four-level socio-ecological paradigm to examine high school resilience. This enables us to analyze and explore the operations at each level, inside and between interconnected systems at the individual, family, school, and community levels. In this study, the microsystem refers to the individual resilience of high school students, which includes levels of goal-planning, help-seeking, family support, affect control, and positive thinking. The mesosystem refers to family resilience, including family belief systems, organizational patterns, and communication. For Bronfenbrenner’s, students are not passive recipients of experiences in these environments but rather people who interact with others and help to structure the environment. Exo-systems refer to school resilience and include the concerns, commitments, dedications, and attitudes of school personnel concerning the educational paths and perspectives of young people, infrastructure, safety, etc. Schools also include connections between microsystems, such as those between families and school experiences and between families and peers. Bronfenbrenner’s argues that strong supportive connections between microsystems may optimize the development of individual student resilience. Macro-systems refer to community resilience, which includes dealing with the promotion of opportunities and collective trust, the promotion of shared values and protection, and the promotion of inter-community trust and relationships. Each system has its dynamics, rules, discourses, and relationships.

Furthermore, according to the multiple resilience theory, the resilience of adolescents is influenced by various interconnected systems. Multi-system resilience is a recently developed concept that recognizes the interconnectedness of several systems in enhancing individual resilience. According to Theron et al. (2022), the foundation of this theory is that individual characteristics do not solely determine resilience but are also impacted by biological, psychological, social, and environmental factors. A multi-system process with interconnected biological, social, institutional, and ecological components is the best way to understand resilience, according to Ungar et al. (2021). Researchers employed a multi-system approach to investigate the elements that contribute to the development of resilience in migrating adolescents. They compared the process to photosynthesis in green plants to demonstrate how individual traits, connections, and broader social institutions generate resilience in migratory teenagers (Wu and Ou, 2021). Research suggests that multi-system resilience influences children’s resilience development (Masten et al., 2016). This means that a child’s behavioral resilience depends on the functioning and interactions of their internal systems (immune system, stress-responsive system, etc.), interindividual or familial resilience, and the broader socio-cultural and ecological systems in which they grow and develop. Individual factors that can contribute to resilience in children who have experienced abuse include cognitive reappraisal, high rumination, high distress tolerance, low expression of aggression, low suppression of emotion, and a secure attachment, according to research by Ungar and Theron (2020). Moreover, on a broader societal scale, elements such as assistance from extended relatives, family unity, parents’ active participation, favorable parenting methods, and household earnings can also impact resilience. In addition, the social support a youngster receives from their community members might influence their behavioral and psychological success. However, multisystemic resilience is crucial for fostering and enhancing individual resilience (Ungar et al., 2023). Research must consider socio-ecological and multi-system views to gain a comprehensive understanding of individual resilience and enhance it. This viewpoint facilitates comprehension of an individual’s encounters and the assistance networks contributing to their ability to bounce back from adversity (Masten, 2021). Using this strategy, family, school, and community workers can efficiently offer children and adolescents the essential care and support they need to flourish and develop resilience.

To summarize, Bronfenbrenner’s ecosystem theory and multisystem resilience theory are two concepts that provide insights into the dynamics of individuals’ interactions with their surroundings. Bronfenbrenner’s ecosystems are based on how people interact with their surroundings, which comprise many smaller systems that affect how they grow and change. It emphasizes the significance of considering the broader framework of an individual’s growth and the interplay between many systems. Multisystem resilience theory is an extension of ecosystem theory that examines the ability of individuals and systems to adapt and flourish in the presence of difficulties. It highlights the significance of comprehending the intricate interplay of multiple systems and their collaborative efforts in fostering resilience. This theory also emphasizes the influence of culture and environment on the development of an individual’s resilience. Bronfenbrenner’s ecosystem and multisystem resilience theories are crucial conceptual frameworks for comprehending the intricate dynamics between people and their surroundings and how these dynamics impact individual resilience.

## 4 Materials and methods

### 4.1 Participants

In this study, we maintained internal and external validity by implementing rigorous sample selection procedures and considering multiple aspects to create a sample that accurately represents the population. Concretely, we enlisted individuals from five high schools (comprising all three high school grades) located in central China, aged 14–20 years old. The age range was meticulously chosen to encompass several age groups of high school students, encompassing a pivotal phase of their academic and social growth. The sample had a mean age of 16.25 years with a standard deviation of 1.868 (*M* = 16.25, SD = 1.868). This information is crucial for understanding the average age and the spread of ages in the sample, ensuring its diversity. To ensure diversity and representation, gender, area (rural and urban), and grade level were evaluated during sample selection. This enhanced the external validity of the findings, hence increasing the study’s conclusions’ generalizability and applicability to adolescents across all genders, locations, and grade levels. In order to maintain the study’s precision and dependability, individuals with cognitive disorders and language problems were deliberately excluded. These circumstances could undermine their capacity to respond accurately to the assessment items. Using this exclusion criterion, we got consistent results from our sample, improving our understanding of the study questions. The precise delineation and justification of these criteria for selecting samples were fundamental components of the study and contributed to guaranteeing the study’s excellence and dependability. Out of the remaining participants, there were 339 (50.8%) female high school students and 328 (49.2%) male high school students. In addition, there were 338 high school students (50.7%) from urban areas and 329 high school students (49.3%) from rural areas. There were 172 (25.8%) first-year high school students, 323 (48.4%) second-year students, and 172 (25.8%) seniors.

The study was performed during May and June 2023. We notified the school principals, parents, and legal guardians regarding the study and acquired their explicit agreement. The study respondents were also notified and requested to provide verbal consent. The voluntary nature of participation and the strict confidentiality of all data were explicitly stated. Furthermore, it is essential to emphasize that the questionnaires included in this study were filled out online in a manner that guaranteed the confidentiality of the participants. Since no personally identifiable information was collected, there was no risk to individuals’ safety and privacy.

### 4.2 Measurements

#### 4.2.1 The resilience scale for Chinese adolescents (RSCA)

The Resilience Scale for Chinese Adolescents (RSCA) was developed in China (Hu and Gan, 2008). The scale consists of 27 items that assess five distinct factors: goal planning (e.g., “I possess a clear sense of purpose in life”), help-seeking (e.g., “I tend to internalize my thoughts rather than seeking support from others”), family support (e.g., “My parents valued my perspective”), Emotional regulation (e.g., “I possess the ability to manage my emotions within a brief timeframe effectively”) and Optimistic mindset (e.g., “I believe that every situation has its advantageous aspects”) Furthermore, the scale encompasses a total of 12 components that are rated in the opposite direction. The responses were given using a Likert-type scale that ranged from 1 (indicating complete disagreement) to 6 (indicating entire agreement). Gan and Yu (2011) demonstrated that the most effective method for assessing the resilience level of Chinese teenagers is by applying the overall score of the Resilience Scale, which incorporates several dimensions of resilience. Chen (2019) confirmed this conclusion. Based on this discovery, this study will employ the overall psychological resilience score to characterize the level of psychological resilience among Chinese high school students. The current study yielded Cronbach’s alpha coefficients of 0.968 for the overall RSCA, 0.890 for goal planning, 0.860 for help-seeking, 0.873 for family support, 0.908 for affect management, and 0.859 for optimistic thinking.

#### 4.2.2 The Chinese family resilience scale (C-FRS)

Family resilience in Chinese communities can be measured using the 35-item Chinese Family Resilience Scale (C-FRS; Leung et al., 2023). Each item was evaluated using a six-point Likert scale, ranging from “strongly disagree” (1) to “strongly agree” (6). More resilient families had higher mean scores. The scale comprises three dimensions: family belief system (e.g., “Family members hold the belief that “adversities” contribute to personal growth”), family organizational patterns (e.g., “Family members can adapt and assume additional tasks and responsibilities when necessary”), and family communication (e.g., “Family members demonstrate proficiency in discussing problem-solving strategies”). This rating is a fair way to measure how resilient Chinese families are. The current study found that Cronbach’s alpha coefficient was 981 for the entire C-FRS, 0.950 for the family belief system, 0.954 for the family organizational patterns, and 0.952 for family communication.

#### 4.2.3 The resilience scale of schools—Youth version (RSS-Y)

A Chinese version of the School Resilience Scale for adolescents has yet to be created in China. Consequently, this investigation employed the Resilience Measure of Schools—Youth Version (RSS-Y) measure, which Milheiro and Marques created (Milheiro Silva and Marques da Silva, 2022). The questionnaire was translated into Chinese using the back translation method. In order to guarantee precision, two proficient English educators from a secondary educational institution were enlisted to evaluate and amend the questionnaire. The questionnaire items were assessed using a six-point Likert scale ranging from 1 (indicating severe disagreement) to 6 (indicating strong agreement). The assessment comprises 17 items evaluated using a six-point Likert scale to ascertain the level of agreement. The scale primarily encompasses three factors: Factor 1 has five elements that represent the worries, commitments, dedications, and attitudes of school personnel toward the educational courses and viewpoints of young individuals. Factor 2 comprises eight items. The issue revolves around the viewpoints of adolescents regarding educational strategies aimed at fostering inclusivity and active engagement, facilitating the expression of opinions, and nurturing the growth of analytical thinking skills. Three components make up Factor 3, which represents students’ views on school facilities, safety, and resources. The current investigation yielded a Cronbach’s alpha coefficient of 0.969 for the entire RSS-Y scale, 0.903 for factor 1, 0.924 for factor 2, and 0.925 for factor 3.

#### 4.2.4 The community resilience scale for youth (CRS-Y)

A Chinese version of the Community Resilience Scale for Adolescents has yet to be created and established in China. The study employed the Community Resilience Scale for Youth (CRS-Y), a measurement tool created by Silva et al. (2022), to gather data. The back translation translated the questionnaire into Chinese. In addition, two highly skilled English educators from a secondary school were invited to assess and revise the questionnaire. Each item was evaluated using a six-point Likert scale, where a rating of 1 represented “strongly disagree” and a rating of 6 represented “strongly agree.” A more excellent mean score signifies a more resilient community. The Community Resilience Scale for Youth (CRS-Y) was created to assess the perceptions of young individuals regarding resilience as a positive aspect of personal growth and a significant encounter within local communities. The scale comprises three components and 12 items. Factor 1 comprises five components that pertain to the promotion of opportunities and the establishment of communal trust. Factor 2 comprises four components pertaining to promoting and safeguarding shared values. Factor 3 consists of three elements that relate to promoting trust and relationships within the community. The current investigation yielded Cronbach’s alpha coefficients of 0.946 for the entire CRS-Y scale, 0.918 for factor 1, 0.915 for factor 2, and 0.746 for factor 3.

### 4.3 Analytical strategy

We evaluated the internal consistency of the instruments by computing Cronbach’s alpha coefficients for each scale. Furthermore, we computed the adjusted total score correlations for each element inside each scale to evaluate their impact on the internal coherence of the scales. In order to examine the association between the resilience levels of Chinese high school students and the resilience scores of their family, school, and community, we computed Pearson correlation coefficients for individual resilience, family resilience, school resilience, and community resilience. Then, we tried to use regression analysis to examine how high school students’ resilience was attached to their school, family, and community resilience. In addition, we employed *t*-tests and multivariate ANOVA to assess the average scores of various groups (such as boys and girls, urban and rural areas, and different grade levels) on the resilience scale. By employing this approach, we evaluated distinctions in resilience among high school students according to gender, geographical location, and academic year. The statistical significance level was established at *p* < 0.05. Cohen’s d and partial η^2^ were computed as effect size measures. We performed descriptive analyses with the SPSS 24 program.

## 5 Results

### 5.1 Descriptive analysis, correlations, and regression statistics

An exploratory approach was employed to assess resilience in Chinese high school students across individual, family, school, and community domains. The analytical findings are presented in Table 1.

**TABLE 1:** Descriptive analysis and correlations.

		M	SD	α	2	3	4	5	6	7	8	9	10	11	12	13	14	15	16	17	18
1	*RSCA-factor 1*	3.266	1.143	0.89	0.682**	0.680**	0.839**	0.863**	0.855**	0.827**	0.896**	0.842**	0.813**	0.797**	0.866**	0.864**	0.635**	0.902**	0.895**	0.848**	0.874**
2	*RSCA-factor 2*	3.228	1.161	0.86		0.855**	0.738**	0.656**	0.598**	0.591**	0.635**	0.592**	0.561**	0.585**	0.589**	0.579**	0.382**	0.862**	0.633**	0.618**	0.581**
3	*RSCA-factor 3*	3.22	1.138	0.873			0.771**	0.693**	0.636**	0.625**	0.664**	0.623**	0.593**	0.609**	0.616**	0.600**	0.390**	0.878**	0.668**	0.644**	0.604**
4	*RSCA-factor 4*	3.293	1.139	0.908				0.902**	0.845**	0.824**	0.876**	0.834**	0.809**	0.794**	0.842**	0.835**	0.571**	0.948**	0.883**	0.852**	0.839**
5	*RSCA-factor 5*	3.278	1.167	0.859					0.873**	0.845**	0.905**	0.854**	0.834**	0.806**	0.886**	0.875**	0.623**	0.921**	0.910**	0.857**	0.885**
6	*CFRS-factor 1*	3.33	1.125	0.95						0.874**	0.900**	0.854**	0.972**	0.806**	0.880**	0.874**	0.681**	0.850**	0.961**	0.861**	0.894**
7	*CFRS-factor 2*	3.188	1.068	0.954							0.883**	0.835**	0.890**	0.769**	0.851**	0.852**	0.664**	0.828**	0.956**	0.828**	0.868**
8	*CFRS-factor 3*	3.286	1.145	0.952								0.932**	0.866**	0.869**	0.925**	0.912**	0.702**	0.887**	0.967**	0.933**	0.934**
9	*RSSY-factor 1*	3.307	1.168	0.903									0.823**	0.843**	0.880**	0.866**	0.686**	0.836**	0.910**	0.934**	0.892**
10	*RSSY-factor 2*	3.317	1.114	0.924										0.768**	0.847**	0.847**	0.674**	0.806**	0.944**	0.824**	0.866**
11	*RSSY-factor 3*	3.173	1.185	0.925											0.857**	0.829**	0.673**	0.801**	0.849**	0.955**	0.864**
12	*CRSY-factor 1*	3.242	1.187	0.918												0.922**	0.700**	0.849**	0.922**	0.899**	0.974**
13	*CRSY-factor 2*	3.24	1.22	0.915													0.700**	0.838**	0.915**	0.875**	0.967**
14	*CRSY-factor 3*	3.111	0.851	0.746														0.581**	0.711**	0.709**	0.806**
15	*RSCA*	3.259	1.04	0.968															0.890**	0.852**	0.845**
16	*CFRS*	3.266	1.069	0.981																0.911**	0.936**
17	*RSSY*	3.227	1.128	0.969																	0.909**
18	*CRSY*	3.209	1.046	0.946																	

**Correlation is significant at the 0.01 level (two-tail). RSCA-factor 1, goal planning; RSCA-factor 2, help-seeking; RSCA-factor 3, family support; RSCA-factor 4, affect control; RSCA-factor 5, positive thinking; CFRS-factor 1, family beliefs system; CFRS factor 2, family organizational pattern; CFRS-factor 3, family communication; RSSY-factor 1, the concerns, commitments, dedications, and attitudes of school personnel concerning the educational paths and perspectives of young people; RSSY-factor 2, the perspectives of young people on school practices to promote inclusion and participation, the expression of opinions, and the development of critical thinking; RSSY-factor 3, the perception of young people about school resources, infrastructure, and safety; CRSY-factor 1, the promotion of opportunities and collective trust; CRSY-factor 2, the promotion of common values and protection; CRSY-factor 3, the promotion of intercommunity trust and relationships; RSCA, The Resilience Scale for Chinese Adolescents; C-FRS, The Chinese family resilience scale; RSS-Y, The Resilience Scale of Schools—Youth Version; CRS-Y, The Community Resilience Scale for Youth.

Table 1 displays the variables’ average values, standard deviation, dependability, and associations. The normalcy and dependability values were considered satisfactory, with an alpha value greater than 0.70. The additional factors identified in the RSCA model are goal planning, help-seeking, family support, affect control, and optimistic thinking. CFRS-factor 1 refers to the family’s belief system; CFRS-factor 2 represents the family’s organizational pattern; and CFRS-factor 3 relates to the family’s communication. The RSSY-factor 1 refers to the worries, commitments, dedications, and attitudes of school personnel toward young people’s educational routes and viewpoints. RSSY-factor 2 refers to the viewpoints of young individuals toward school strategies that foster inclusivity and engagement, encourage the expression of opinions, and cultivate critical thinking skills. RSSY-factor 3 refers to the view of school resources, infrastructure, and safety as young individuals perceive. CRSY-factor 1 refers to the facilitation of opportunities and the cultivation of communal trust. CRSY-factor 2 refers to advancing shared principles and safeguarding, whereas CRSY-factor 3 pertains to enhancing trust and connections between different communities.

Besides, correlation analysis assesses the linear correlation between two variables, quantifying the strength and direction of the association (Crawford, 2006). The correlation coefficient (r) quantifies the extent of correlation in a linear relationship between the two variables. A positive correlation indicates a direct relationship between two variables, meaning that if one variable increases, the other also increases. A value of 1 indicates a strong association. A value of −1 indicates a perfect negative linear correlation. Most people accept that the correlation is weak if *r* < 0.4, moderate if 0.4 < r < 0.8, and vigorous if r > 0.8 (Shi and Conrad, 2009).

Analyzing the correlation coefficients from Table 1, it can be seen that all variables have correlations, and all variables are positive. Of these factors, CRSY-factor 3 and RSCA-factor2 exhibit low correlations, while CRSY-factor 3 and RSCA-factor 3 also have low correlations. Every other factor has either a moderate or strong relationship. Correlation serves as empirical support for a connection between two variables. Nevertheless, the presence of one variable does not necessarily imply causation by another. This duty is delegated to regression. Regression relies on the premise that the researcher must initially possess a legitimate justification to posit a cause-and-effect connection between two or more variables (Costa, 2017). The researcher proceeds to utilize regression analysis to investigate the correlation between the dependent variables (resilience of individual high school students) and the independent factors (resilience of high school students in terms of school, family, and community). The analysis results are shown in Table 2.

**TABLE 2:** Summary table of multiple linear regression analysis of high school students’ individual resilience to family resilience, school resilience, and community resilience.

Sequence of variables for prediction	*R*	*R* ^2^	*R*^2^ adjusted	*F*	β
1. Community resilience	0.806	0.649	0.649	1760.164***	0.681
2. Family resilience	0.840	0.705	0.704	1348.639***	0.015
3. School resilience	0.842	0.709	0.707	897.872**	0.246

Correlation is significant at the 0.01 level (two-tail).

***Correlation is significant at the 0.001 level (two-tail); *R*, multiple correlation coefficient; *R*^2^, multivariate regression determination coefficient; β, beta values.

This study investigated the impact of family, school, and community resilience on the individual resilience of high school students by utilizing multiple linear regression models. An R-squared value of 0.709 indicated that the regression model fit well. This value suggests that the three independent variables accounted for 70.9% of the variability observed in the resilience of high school pupils. The regression model had a high significance level, evidenced by an *F*-value of 897.872 and a *p*-value of less than 01 (*p* < 0.01). This indicates that the regression model outperformed the null model, which lacks independent variables. The regression coefficient table reveals that family, school, and community resilience are significant predictor variables. The β-value represents their standardized regression coefficients, indicating the number of standard deviations by which the dependent variable will change for every standard deviation change in the independent variable while keeping the other independent variables constant. Based on the β-values, it is clear that community resilience (0.681) has the most significant impact on high school students’ individual resilience compared to school resilience (0.246) and family resilience (0.015). These findings suggest that community resilience has a significant role in shaping the resilience of high school students, whereas family resilience has a minimal impact. The study’s findings suggest that enhancing the resilience of high school students may be achieved by focusing on creating community environments and increasing the availability of community services and support. Therefore, H1 research is valid.

### 5.2 Individual, family, school, and community resilience among high school students based on a variety of individual characteristics

#### 5.2.1 Gender dimension

*T*-tests were conducted to examine gender differences on the four scales. Results from the Independent Samples *T*-test showed that, in terms of gender, girls’ resilience was significantly higher than boys’ resilience (*p* < 0.001) (as shown in Table 3). As a result, Chinese high school girls outperform their male counterparts in terms of resilience across all domains: individual, institutional, familial, and community. The Cohen’s d analysis revealed statistically significant gender disparities across all scales. The observed disparities were substantial, signifying that females exhibited much superior scores on average compared to males.

**TABLE 3:** Mean scores, standard deviations, and differences in individual, family, school, and community resilience by gender.

Scale (*N* = 667)	Girl (*N* = 339)		Boy (*N* = 328)		Gender difference	
	M	SD	M	SD	Cohen’s d	T
RSCA	3.722	0.936	2.781	0.919	−1.015	−13.098***
CFRS	3.766	0.989	2.750	0.889	−1.079	−13.933***
RSSY	3.667	1.091	2.772	0.978	−0.863	−11.141***
CRSY	3.671	0.982	2.731	0.883	−1.005	−12.975***

***Correlation is significant at the 0.001 level (two-tail); RSCA, The Resilience Scale for Chinese Adolescents; C-FRS, The Chinese family resilience scale; RSS-Y, The Resilience Scale of Schools—Youth Version; CRS-Y, The Community Resilience Scale for Youth.

#### 5.2.2 Region dimension

The findings from the Independent Samples *T*-test indicate that there was a significant difference in the level of resilience between urban and rural high school students, specifically about the location of their families’ residences (*p* < 0.001) (as shown in Table 4). Moreover, a notable observation was that urban high school students attained higher scores and had exceptional resilience in academics, family, and community. Furthermore, Cohen’s d values suggest a notable regional disparity across all scales, with urban high school students exhibiting much higher average scores than their rural counterparts.

**TABLE 4:** Mean scores, standard deviations, and differences in individual, family, school, and community resilience by region.

Scale (*N* = 667)	Urban (*N* = 338)		Rural (*N* = 329)		Region difference	
	M	SD	M	SD	Cohen’s d	T
RSCA	3.783	0.930	2.720	0.857	−1.188	−15.341***
CFRS	3.856	0.922	2.660	0.849	−1.349	−17.419***
RSSY	3.764	1.062	2.675	0.978	−1.103	−14.234***
CRSY	3.748	0.961	2.655	0.883	−1.225	−15.810***

***Correlation is significant at the 0.001 level (two-tail); RSCA, The Resilience Scale for Chinese Adolescents; C-FRS, The Chinese family resilience scale; RSS-Y, The Resilience Scale of Schools—Youth Version; CRS-Y, The Community Resilience Scale for Youth.

#### 5.2.3 Grade dimension

The chi-square test showed that there were significant differences in individual, family, school, and community resilience (*P* < 0.01) between the different groups of adolescents (as shown in Table 5). There are substantial disparities across all levels of measurement. The results obtained from the LSD *post-hoc* test indicate a negative correlation between grade level and resilience ratings among high school students. This relationship is applicable at the individual, family, school, and community levels. As adolescents progress to higher grade levels, their ability to cope with challenges and bounce back from adversity in their personal lives, family dynamics, school environment, and community tends to decrease. According to results of the study, a student’s performance tends to peak in their first year, decline in their sophomore year, and bottom out in their senior year. Additionally, partial η^2^ values showed significant grade discrepancies across all scales.

**TABLE 5:** Mean scores, standard deviations, and differences in individual, family, school, and community resilience by grade.

Scale (*N* = 667)	1. H.S.1 (*N* = 172)		2.H.S.2 (*N* = 323)		3.H. S.3 (*N* = 172)		Grade difference		
	M	SD	M	SD	M	SD	Partial η^2^	F	LSD
RSCA	3.837	0.797	3.207	1.101	2.779	0.854	0.205	52.149***	1 > 2 > 3
CFRS	3.953	0.726	3.232	1.125	2.644	0.824	0.226	79.886***	1 > 2 > 3
RSSY	3.838	0.724	3.217	1.197	2.634	1.002	0.157	57.261***	1 > 2 > 3
CRSY	3.857	0.678	3.196	1.108	2.583	0.821	0.202	78.524***	1 > 2 > 3

***Correlation is significant at the 0.001 level (two-tail); RSCA, The Resilience Scale for Chinese Adolescents; C-FRS, The Chinese family resilience scale; RSS-Y, The Resilience Scale of Schools—Youth Version; CRS-Y, The Community Resilience Scale for Youth.

## 6 Discussion

This study expands upon Bronfenbrenner’s ecosystem and multisystem resilience theories, positing that social ties influence children’s and adolescents’ resilience, behaviors, and beliefs. The interplay between people and their environment, which Bronfenbrenner’s argues is a complex web of interdependent systems, is a critical component of human growth (Chen and Song, 2022). Multisystem resilience theory emphasizes understanding the intricate relationships between multiple systems and how they work together to promote individual resilience (Masten, 2021). Multisystem resilience theory builds upon Bronfenbrenner’s ecosystem hypothesis by examining the interplay among several systems and their role in generating individual resilience when facing challenges. This encompasses an individual’s immediate surroundings and the broader cultural and social milieu in which they reside. Multisystem resilience theory aims to discover characteristics that facilitate individual resilience and enable individuals to adapt and flourish in adversity by comprehending the intricate interconnections across various systems. Bronfenbrenner’s ecosystem and multisystem resilience theories highlight the significance of the interplay among several systems in nurturing individual resilience. These ideas suggest that individuals are members of families, schools, communities, and peer groups. Consequently, these familial, educational, communal, and social contexts influence the formation of personal resilience. The results of this investigation align with these beliefs. The study revealed that strong connections to family and school, along with the benefits and incentives provided by these environments and, to a lesser degree, positive behaviors in the community, can significantly contribute to the cultivation and growth of resilience in high school children.

### 6.1 A general description: individual, family, school, and community resilience in high school students

This study found that Chinese high school students score lower on individual, family, school, and community resilience than the midpoint of a six-point scale. Family resilience is the highest ranked of the four dimensions, followed by individual, school, and community resilience. The study’s conclusions are intricately connected to China’s cultural values. China has consistently prioritized the communal ethos of the family and the notion of common principles throughout its history. In Chinese culture, the family unit is highly valued and considered the cornerstone, as it involves incorporating individuals into the collective and harmonizing their objectives with the welfare of the family, society, and country. This is apparent in multiple facets of Chinese society, encompassing traditional legal culture, organ donation, work unit structures, and cuisine culture (Hung, 2023). In Chinese culture, the family is regarded as the fundamental unit of “procreation” and the embodiment of “stability,” with the structure of the cosmos and societal existence centered around the notion of “family” (Li, 2019). For most Chinese individuals, the family has assumed a central role in their lives, with kinship as the most potent social connection (Topley and Freedman, 1971). A distinct culture of filial piety develops when parents take an active role in their children’s upbringing and education and when children reciprocate by supporting and caring for their parents (Nainee et al., 2016). Consequently, it should come as no surprise that the resilience measured reveals Chinese students in high school to have the most resilient families.

### 6.2 Correlation analyses of high school students’ individual resilience and family resilience, school resilience, and community resilience

This research examined the correlation between the individual resilience of Chinese high school students and the resilience of their families, schools, and communities. The study employed self-report questionnaires to evaluate students’ subjective assessments of their resilience levels. The data analysis revealed a substantial correlation between Chinese high school students’ five psychological resilience factors and the individual factors of family, school, and community psychological resilience. This implies that the student’s judgments of their resilience and the environment’s resilience are in agreement, and it further demonstrates their fulfillment and relationship with these situations. The results are consistent with those of the study conducted by Klocke et al. (2013). The study confirms that variables such as the economy (including GDP and inequality) and education expenditure substantially impact children and adolescents’ resilience and subjective wellbeing. In contrast, the quality of their relationships with their communities, schools, families, and schools significantly impacts their resilience and wellbeing (Ungar et al., 2019). This discovery suggests that the specific conditions experienced by adolescents and kids have a more immediate influence on their ability to bounce back from adversity than broader societal variables. While personal characteristics significantly influence an individual’s ability to bounce back from adversity, the family, educational institution, and broader social milieu can also affect an individual’s life. Various studies have emphasized the significant impact of family, school, and community on enhancing the resilience of children and adolescents (Dvorsky et al., 2020). School and community environments foster positive relationships with adults and peers by actively implementing mentorship programs and providing numerous opportunities for students to form such relationships (Masten et al., 2008). Schools and communities may offer impoverished students essential nourishment and healthcare, supporting optimal brain development, physical growth, and skill acquisition. Communities, schools, and instructors offer daily opportunities to learn from experiences, succeed, and enjoy accomplishments. These opportunities help kids and adolescents develop intrinsic motivation, self-efficacy, and resilience when faced with failure. In addition, research has shown adolescents benefit from having their parents’ support system, which helps them build resilience (Tian et al., 2018) and positively impacts the development of an individual’s internal resources as a protective factor (Kumpfer and Summerhays, 2006). Extensive research suggests that by prioritizing the development of resilience in educational institutions, communities, and families, there is a strong likelihood of improving the individual resilience of kids and adolescents (Tian et al., 2018).

### 6.3 The resilience of high school students’ individual, family, school, and community and their different individual characteristics

Individual, familial, academic, and community resilience among Chinese high school students may vary substantially by gender, region, and grade level, according to the findings of the data analysis.

An initial finding of this study could be that female high school students exhibited superior performance on measures of individual, familial, academic, and community resilience, thereby demonstrating greater capacity and resilience. This could be attributed to the girls’ proficiency in interpersonal connections. According to Crosnoe (2000), girls typically perform better in interpersonal relationships than guys. This can be related to their capacity to form robust emotional connections with their family, teachers, and friends. Moreover, females tend to cultivate more intimate interpersonal connections. Consequently, compared to boys, girls tend to receive more emotional support from their parents, friends, siblings, and teachers (Crosnoe and Elder, 2004). According to Sakhat (2017), providing emotional support can boost girls’ confidence, self-esteem, self-efficacy, and coping abilities, improving their resilience. Conversely, males might be susceptible to the impact of social culture, which may mold their perception of emotional expression as indicative of frailty. Consequently, they may be more likely to repress their emotions and exhibit deficiencies in coping mechanisms and effective emotional regulation. Boys could have had increased feelings of helplessness, depression, and anxiety when confronted with challenges and stress, resulting in a decrease in their resilience (Scheff, 2006). Hence, gender significantly impacts the resilience of high school students.

Furthermore, this study revealed that high school students residing in urban regions have superior resilience in dealing with setbacks compared to their counterparts in rural areas. Adolescents in urban and rural areas may exhibit elevated personal, familial, educational, and societal resilience. This could be associated with urban regions’ living circumstances, educational resources, and social possibilities. Initially, metropolitan infrastructure exhibits a higher level of development, offering more conducive schooling circumstances. Urban schools have superior resources, including enhanced equipment, textbooks, and more proficient educators, compared to their rural counterparts (Glaeser, 2011). Consequently, high school pupils educated in such a setting inherently possess an edge in attaining superior academic results. High school students’ academic success influences their resilience in several ways, including how they feel about themselves and what they hope to do in the future (Yan and Gai, 2022). Further, family variables can strengthen and improve children’s resilience (Walt, 2006). Urban families typically enjoy superior economic circumstances, enabling parents to allocate more significant financial resources, time, effort, and other assets toward nurturing their children (Zhang et al., 2023). These resources may offer additional educational and developmental prospects and increased social and emotional assistance. In addition, urban parents experience greater ease in managing life’s difficulties, offering their children an improved living environment and a more stable family upbringing. Moreover, urban adolescents possess a more significant social advantage than their rural counterparts due to gaps in living conditions and educational environments (Zhao and Zhou, 2020). Cities offer more excellent social options, such as extracurricular cultural classes and specialized training institutions. These opportunities facilitate young people’s capacity to interact with classmates, establish new friendships, and enhance their social aptitude. Furthermore, the educational environment in metropolitan areas is more dynamic, and youngsters are more eager to learn than their rural counterparts. Urban parents are more inclined to allocate time and effort toward their children’s educational development (Jeynes, 2007). Consequently, children living in metropolitan areas exhibit higher motivation, enthusiasm for learning, and academic achievement. The elements above contribute to urban high school students’ intellectual superiority over their rural counterparts and access to better social infrastructure, broader mentalities, increased exposure to new experiences, more robust family support, and more educational options. Urban high school adolescents demonstrate exceptional individual, family, school, and community adaptation talents.

In addition, this study found that there may be a negative correlation between grade level and psychological resilience in high school students at the individual, family, educational, and societal levels. More precisely, the resilience of first-year students was the highest, followed by second-year students, and finally, third-year students. This might be associated with the Chinese method of college entrance examinations. According to the Chinese college entrance test system, pupils who reach their third year of high school receive significant attention and are promptly identified as the “primary support object” by the school, family, and society (Liu and Helwig, 2020). These “support” and “attention” primarily impose tangible and intangible pressures on adolescents. The particular expression of this “support” and “importance” primarily involves the imposition of a substantial academic workload, frequent examinations, and excessive responsibilities on high school students. This frequently results in a broader spectrum of internal conflicts and pressures for these students (Cai et al., 2016; Liu and Helwig, 2020). The pressures and tensions experienced by high school students can undermine their self-identity, self-control, and self-efficacy, ultimately diminishing their resilience. What’s more, the college entrance examination system may also affect the resilience of high school students’ families, schools, and communities. The college admission examination holds significant weight, leading to excessive intervention and supervision by families and schools in the lives and education of high school students. Consequently, high school students need more autonomy and freedom (Zhao et al., 2015). This could undermine trust and communication between high school students and their parents and instructors, thereby diminishing their overall family and school resilience. Additionally, the college admissions examination system might restrict high school student’s social engagements and interests, leading to a diminished sense of connection with and participation in their society. Consequently, this diminishes their ability to adapt to and recover from challenges within their community (Hasan, 2021). Accordingly, the college entrance examination system could significantly contribute to the decrease in resilience among senior students.

## 7 Strengths, limitations, and future directions

This study possesses several strengths. This study seeks to fill a research vacuum by examining the influence of family, school, and community resilience on individual resilience. The results add to what is already known about resilience and strengthen the foundation for future studies in the field. Moreover, it offers theoretical perspectives and practical advice on fostering resilience among high school students. Furthermore, this research provides a new definition of individual resilience in high school students, encompassing resilience across several systems such as family, school, and community. Reframing makes a more pertinent elucidation of the resilience development process possible. It is essential because family, school, and society are big in shaping and building resilience in high school students (Ungar et al., 2019). This novel viewpoint also presents opportunities for future investigations to examine the possible moderating influence of individual resilience in the presence of risk within familial, educational, and communal settings and its potential effect on mental wellbeing. Additionally, our findings could significantly assist parents, guardians, and educators in cultivating the development and advancement of individual resilience in high school students. This might be achieved by utilizing both structured and unstructured instructional methods. Furthermore, this study illuminates neglected variables that may impact the execution of novel strategies to foster resilience development in high school adolescents, specifically for policymakers. Besides, as researchers, we provide factual information and theoretical perspectives that can direct forthcoming inquiries into the resilience of high school children, considering various aspects such as the individual, family, school, and community.

In addition, this study has certain constraints. Initially, the study relied only on high school students’ data. It is possible to introduce bias when data is collected only through self-reporting. Also, given that this study exclusively focused on the resilience of a limited sample of Chinese high school students, the conclusions may need to be generalizable to all Chinese high school students. Moreover, it should be noted that the Resilience in Schools Scale—Youth Version (RSS-Y) and the Community Resilience Scale for Youth (CRS-Y) have not undergone validation procedures in China. Ultimately, the study’s primary utilization of a cross-sectional design necessitates careful consideration of potential limitations. Specifically, this approach cannot evaluate longitudinal changes in variables and does not offer the opportunity to consider the influence of confounding variables (Monnier et al., 2015).

To further explore the implications of this study’s results, future researchers must examine the impact of additional variables, including ethnicity and traditional culture, family, school, and community, on the resilience of high school students. In addition, future studies must investigate the impact of high school students’ resilience on the resilience of their families, school, and community. Conducting these studies will offer researchers and practitioners a more thorough comprehension of the results linked to the resilience levels of high school students in terms of individual, family, school, and community factors, as well as the repercussions of poor resilience. Future researchers are advised to employ various qualitative and quantitative methods, including mixed methods, to obtain a more profound understanding of the factors and processes that contribute to the generation of individual resilience, family resilience, school resilience, and community resilience in high school students. In addition, future investigators might choose a longitudinal research strategy to investigate the correlation between individual, family, school, and community resilience among high school students to address the limitations of cross-sectional research designs. Further, it is crucial to acknowledge that understanding family, school, community, and individual resilience in high school students is still very new in resilience research. Consequently, these ideas must be fine-tuned, and measurement techniques must be improved.

## 8 Conclusion

To summarize, the results of this study suggest that Chinese high school students exhibit lower levels of resilience in various domains, including individual, family, school, and community resilience, compared to the midpoint of a six-point scale. These findings indicate that Chinese high school students possess a limited sense of optimism regarding their resilience and the resilience of their family, school, and community. Moreover, the study uncovers a robust correlation between the resilience of individual high school students and the resilience of their families, schools, and communities. The findings show that four areas affect a high school student’s resilience: personal factors, family dynamics, the school environment, and support from the community. Each high school student’s resilience depends on how resilient their family, school, and district are. These findings augment the current understanding of high school adolescents’ resilience and offer valuable insights into the significant contributions of family, school, and society in contributing to their resilience. It is worth mentioning that in China, the family unit assumes a crucial role in equipping high school students with resilience resources. The family is regarded as the foundational social unit and offers individuals the utmost care and attention. To improve high school students’ resilience, it is necessary to use a holistic strategy that considers the family within the context of the more extensive systems of the school, the community, and society (Li et al., 2018). In addition, cultural traditions also enhance the resilience of high school students in the Chinese environment. Ungar (2008) discovered that how individuals handle and resolve conflicts that arise from their interactions with their cultural and environmental surroundings may profoundly impact the development of their resilience.

This little study covered only some aspects of resilience development in Chinese high school students. Nonetheless, the results shed light on effective strategies for boosting high school students’ resilience. By investigating resilience from multiple angles—students, families, schools, and communities—this study adds to our knowledge of how high school adolescents build and refine this vital life skill. The work presents the following policy suggestions to policymakers: To develop effective resilience promotion initiatives, policymakers must consider the resilience assets of high school students across the domains of family, school, and community, as well as the interconnections between these domains. Initially, it is crucial to note that families are vital in generating resilience resources for Chinese high school students. This may be because the family unit is regarded as the fundamental social unit and gives adolescents the utmost level of care and attention (Ali, 2020). High school students greatly benefit from the support of their families, and educational administrators must encourage family involvement in providing resources that promote resilience among high school adolescents (Ungar et al., 2014). This may encompass services such as parental education, familial counseling, active involvement of the family, and assistance for the family to improve their functioning and unity to promote the resilience of high school students. Moreover, considering that school plays a crucial role in cultivating resilience among high school students, administrators have to offer a conducive environment that promotes the development of resilience in those students. This encompasses creating a pleasant learning environment, prioritizing teacher-student relationships, providing various programs and activities, offering suitable challenges and feedback, and implementing strategies that promote high school students’ engagement and sense of belonging. These efforts aim to cultivate self-confidence, self-esteem, self-efficacy, and self-control among high school students, ultimately enhancing their resilience (Riswantyo and Lidiawati, 2021). Additionally, the community must play a vital role in nurturing the resilience of high school students (Stukas and Dunlap, 2002). Education administrators can provide more significant opportunities for high school students to engage and actively participate in the community. Establishing partnerships with community agencies and organizations, providing opportunities for high school students to get involved with community service and volunteer activities, and increasing high school students’ awareness and use of community resources and networks to develop social skills, social responsibility, social support, and social capital for improving high school students’ resilience are all examples of how this can be accomplished. Finally, culture is a necessary background for high school students’ resilience, and cultural traditions in the Chinese setting also contribute to high school students’ resilience. Ungar (2008) figured out that how an individual navigates and reconciles conflicts emerging from interactions with cultural and environmental contexts may significantly affect the development of his or her resilience. Educational administrators should respect and employ high school students’ cultural traditions and beliefs (Simon et al., 2005). This may involve promoting cultural identity, sensitivity, and diversity among high school students. It also entails offering opportunities for them to explore and value their own and others’ cultures and encouraging the utilization of cultural resources and strategies to navigate difficulties and challenges. By doing so, it aims to facilitate the acculturation process of high school students and contribute to the building of cultural diversity.

## Data availability statement

The raw data supporting the conclusions of this article will be made available by the authors, without undue reservation.

## Ethics statement

The studies involving humans were approved by College of Education Jiangxi Normal University. The studies were conducted in accordance with the local legislation and institutional requirements. Written informed consent for participation in this study was provided by the participants’ legal guardians/next of kin.

## Author contributions

CQ: Conceptualization, Data curation, Investigation, Writing–original draft. NY: Supervision, Writing–review and editing.
